# The Herbarium of Paolo Boccone from the Years 1689–1695 Kept in Wrocław (Poland): Plant Nomenclature, Taxonomy and Biogeography

**DOI:** 10.3390/plants15172703

**Published:** 2026-09-02

**Authors:** Jacek Drobnik, Adam Stebel, Magdalena Grenda-Kurmanow, Gabriel Alziar

**Affiliations:** 1 Department of Pharmaceutical Botany, Medical University of Silesia in Katowice, 41-200 Sosnowiec, Poland; astebel@sum.edu.pl; 2 Faculty of Conservation and Restoration of Works of Art, Academy of Fine Arts in Warsaw, 00-379 Warsaw, Poland; magdalena.grenda@asp.waw.pl; 3 Independent Researcher, 184 rue Côte Vieille, 12120 Cassagnes-Bégonhès, France; entiminae12@gmail.com

**Keywords:** historic herbarium, exsiccate, pre-Linnaean, taxonomy, medicinal plants, biogeography, garden plants, flora

## Abstract

Between 1689 and 1695, Paolo Silvio Boccone (1633–1704), an Italian botanist, compiled an herbarium containing 134 plants (or organisms that were considered to be plants at the time), which is currently stored in Wrocław, Poland. The collection mainly comprises species from Sicily and other locations within the central Mediterranean area, as well as some plants from Central and Western Europe. Boccone’s motivation for creating this collection was to seek out rare or unknown plants, both wild and kept in gardens. For the first time, we have identified the plants taxonomically and provided a transcription of their polynomial names (as basonyms of the species) and their geographical locations, as indicated on the original labels in the herbarium. A map of plant locations is provided. Boccone recorded certain plants (introduced later into cultivation or imported to Europe in later centuries) merely as ornamentals kept in gardens. By querying the pharmaceutical literature from the 18th and 19th centuries, we have demonstrated that this is precisely how these exotic rarities came to be recognised as medicinal or industrial plants. Boccone also discovered the rare species *Narthecium reverchonii* Čelak, which is endemic to Corsica.

## 1. Introduction

The tradition of creating herbaria, or collections of dried and described plants, dates back almost 500 years. Professor Luca Ghini (1490–1556) of the University of Bologna in Italy is widely recognised as a pioneer in this field. In the first half of the 16th century, he and his students, Andrea Cesalpino and Ulisse Aldrovandi, developed a method for pressing, drying and mounting plant specimens. Other herbaria soon followed, which were called dry gardens (*hortus siccus* in Latin) or living herbaria (*herbaria viva*) because they contained real plants rather than woodcuts or drawings of them. Herbaria served various purposes. They were used as teaching aids and, due to their often-elaborate design, were given as exclusive and fashionable gifts [1]. The first scientific institution to establish an herbarium was the University of Bologna in Italy in 1568. Over time, herbaria containing increasingly detailed descriptions of individual specimens also began to serve scientific purposes, becoming an indispensable source of knowledge about not only plant species, but also phytogeography and *materia medica* (medicinal herbal materials) [2,3]. The use of herbaria in scientific research led to the establishment of natural history departments at universities, which began appearing as early as the 18th century [4,5]. This method continued for over 200 years. Three collections have now exceeded 8 million specimens each (they are at the Naturalis Biodiversity Center in The Netherlands, Royal Botanic Gardens in Kew, and Muséum National d’Histoire Naturelle in Paris) [6]. However, by the end of the 20th century, it seemed that the traditional role of herbaria was slowly becoming a thing of the past, with them even beginning to be identified as part of troublesome and fragile museum collections usable at most for teaching [7].

Changes in the approach to the potential use of herbaria and their role in modern science have emerged alongside new research tools, methods, and their ever-growing prospects for application. The first innovations were the digitisation and database creation of collections, which took place in the first two decades of the 21st century [8]. Nowadays, herbarium specimens can be used to study long-term trends in plant biotic interactions [9]. Dried plants become a source of genetic material for research on genetic diversity, phylogeny, systematics and evolution of plants [10,11,12,13]. An herbarium is an excellent and handy source of plant material for taxonomic research in situations where species are rare or scattered and difficult to obtain from nature at one time [10]. They also provide information for documentation, planning, and conservation activities for endangered species, constituting a unique, detailed and well-documented record of knowledge about changes in biodiversity over time and space [5,14,15,16,17,18]. Perhaps the genetic material and diaspores obtained from herbaria will be used to restore plant species now extinct in the wild [19,20].

The digitisation of collections plays a vital role in accessing the data contained on labels and the appearance of specimens, enabling, like never before, convenient access to many, often little-known herbarium collections located in the farthest corners of the world and bearing witness to ancient, little-known journeys or the colonial past [9,21,22]. This allows for the assessment of the state of conservation of local and global biodiversity in a spatial and historical approach.

It is predicted that in the near future, integrated herbarium resources will constitute a knowledge bank, already called the “herbarium of the future” or “global metaherbarium” and designed as a research tool for the next half century and beyond [23].

In the context outlined above, regarding the data contained in previously unknown herbaria, especially old ones that are difficult to access, their scientific assessment and publication are part of a global trend. Without a doubt, an example of such scientific objects is the plant collection created by Italian botanist Paolo Silvio Boccone, dating back to the end of the 17th century, currently stored in Wrocław (Poland), and being the oldest herbarium object in this country.

### 1.1. Paulo Silvio Boccone’s Herbaria and Their Monographs

Historians and botanists have catalogued 16 existing Boccone’s herbaria (stored in France, Italy, Poland, England, Holland and Austria) and two others in Italy, now destroyed or lost. The detailed list of these objects and holding institutions has been compiled by [24].

This resource article presents the late 17th-century herbarium of Paulo Boccone, his last unexplored taxonomically collection to survive in Europe. We reveal the historical nomenclature of the plants, and divulge polynomial phytonyms established there by Boccone. This herbarium was created between 1689 and 1695, and it also includes two specimens from the year 1674. Its history, datation, structure, and condition as a historical object were published by [25]. We will refer to this herbarium as the Wrocław collection. The creator of this historical artefact is Paolo Silvio Boccone (1633–1704), an Italian monk, physician and traveller. The herbarium volume measures 41 cm tall, 26 cm wide, and 8–13 cm thick. It has 145 pages (142 numbered), and no title page. It is bound in parchment with cardboard boards, sewn and featuring headbands. The specimens are mounted with paper strips or by overall adhesion. There are (1–)2–3(–5) specimens mounted on each page, and each has an individual label mounted to the sheet [25].

### 1.2. Aims of the Work

The aims of this work are: (1) to list the plant taxa and localities contained in this collection; (2) to divulge Boccone’s original polynomials attached to the specimens gathered by him as a valuable source of his original plant nomenclature (which varied considerably in his other publications); and (3) to provide taxonomical, floristic and biogeographical data useful for studying the history of both wild and horticultural medicinal and other economic plants.

## 2. Results

### The Content of the Collection and the Taxonomic Identification of the Plant Specimens

Handwriting includes plant signatures and pagination. Signatures were made on paper labels of irregular width, which are mounting strips for the specimens. Some of the signatures of the specimens are made directly on the sheets.

The list below presents Boccone’s plant polynomials and locations in his herbarium stored at Wrocław University (Poland), with taxonomical identification of plants.

Formatting and sequence of information are as follows: sheet number, *original name*, *original location*, **taxonomical identification**.

Abbreviations used are: cf. = probably, similar to; Germ. = German; Lat. = Latin; ? = uncertain; and / = or. Necessary additions to transcribed text and our translations are enclosed in [].

Sheet 1: *Filix Indica Osmunda facie Bodaei a Stapei*; location: *ex India*. Identified as ***Onoclea sensibilis* L.**

Sheet 2 top: *Cortusa Americana elegantissima*. Taxon: ***Heuchera americana* L.**?/***H. sanguinea* Engelm.**?

Sheet 2 botttom: *Caryophyllus montanus Rubeolae foliis*; location: *ex Anglia. 1674 collectu*[*s*]. Taxon: ***Dianthus deltoides* L.**?/***D. gratianopolitanus* Vill.**?

Sheet 3: *Gossypium Matthioli*. Taxon: ***Gossypium arboreum* L.**

Sheet 4 top: *Papaveri affinis montana Aconiti folio Pauli Hermanni Mentzelii*; location: *ex America*. Taxon: ***Podophyllum peltatum* L.**

Sheet 4 bottom: *Alsine maritima spergulae facie media Bauh. Pin.*; location: *ubiq*[*ue*]. Taxon: ***Spergularia marina* (L.) Besser**

Sheet 5: *Daucus Creticus verus Matthioli Bauhini*; location: *Panormus*. Taxon: ***Athamanta cretensis* L.**

Sheet 6 top: *Gramen filiceum paniculis integris Bocconi*; location: *Panormi*. Taxon: ***Desmazeria sicula* (Jacq.) Dumort.**

Sheet 6 bottom: *Litospermum umbellatum latifolium Bocconi*; location: *Panormi*. Taxon: ***Glandora rosmarinifolia* (Ten.) D.C.Thomas**

Sheet 7: *Cervicaria erecta*; location: *ex Bononia*. Taxon: ***Campanula bononiensis* L.**

Sheet 8 top: *Saponaria sphoerula*; location: *ex India*. Taxon: ***Sapindus saponaria* L.**

Sheet 8 middle: *Anchusa echioides lutea Fab*[*ricii*] *Columnae*; location: *Sicula*. Taxon: ***Onosma echioides* L.**

Sheet 8 bottom: *Ancusa Cretica Prosp.*[*eri*] *Alpini*; location: *Sicula*. Taxon: ***Anchusa cretica* Mill.** (= *Anchusella cretica* (Mill.) Bigazzi, E.Nardi & Selvi)

Sheet 9 top: *Astragalus hispanicus siliqua epiglotidi simili flore albo minor Turnefortii*; location: *ex Sicula*. Taxon: ***Biserrula epiglottis* (L.) Coulot, Rabaute & J.-M.Tison**

Sheet 9 bottom: *Heleborus trifoliatus*; location: *ex Corsica*. Taxon: ***Helleborus argutifolius* Viv.**

Sheet 10 top: *Trifolium stellatum Bauh. Prodr.*; location: *ex Sicilia*. Taxon: ***Trifolium stellatum* L.**

Sheet 10 bottom: *Sena*; location: *ex Egypto*. Taxon: ***Senna italica* Mill.**

Sheet 11 top: *Heliochrysum sylvestri tomentoso simile*; location: *in Italia*. Taxon: ***Phagnalon rupestre* (L.) DC.**

Sheet 11 bottom: *Serpentina crasso folio*; location: *Neapolitana*. Taxon: ***Plantago serpentina* All.** or ***P. crassifolia* Forssk.**

Sheet 12 top: *Solanum spinosum pomiferum Aphricanum*; location: *ex Africa*. Taxon: ***Solanum linnaeanum* Hepper & P.-M.L. Jaeger**

Sheet 12 bottom: *Solanum spinosum maxime tomentosum Boccon.*; location: *ex America*. Taxon: ***Solanum tomentosum* L.**

Sheet 13 top: *Solanum Indicum incanum non spinosum*; location: *ex America*. Taxon: ***Solanum incanum* L.**

Sheet 13 middle: *Sedum alpinum aiugae folio*; location: *ex Sabaudia*. Unidentifiable.

Sheet 13 bottom: *Sedum alpinum Dryphis folio*; location: *ex Sabaudia*. Taxon: ***Draba aizoides* L.**

Sheet 14 top: *Hypericum hispanicum glabrum*; location: *in montibus Italiae*. Taxon: ***Hypericum perfoliatum* L.**

Sheet 14 middle: *Gallium siculum incanum*. Taxon: ***Galium* cf. *lucidum* All.**

Sheet 14 bottom: *Ascyrum magnoflore Bauh. Pin.*; location: *in Sicilia*. Taxon: ***Hypericum calycinum* L.**

Sheet 15 top: *Trifolium aspaltites sive Bituminosum*; location: *in litore Italiae*. Taxon: ***Bituminaria bituminosa* (L.) C.H. Stirt.**

Sheet 15 bottom: *Convolvulus siculus minor flore parvo auriculato Bocconi*. Taxon: ***Convolvulus siculus* L.**

Sheet 16: *Urtica prima sive Romana*. Taxon: ***Urtica pilulifera* L.**

Sheet 17: *Polygonatum angustifolium*; location: *Siculum*. Taxon: ***Polygonatum multiflorum* (L.) All.**

Sheet 18 top: *Convolvulus siculus supinus oleae folio argenteus*. Taxon: ***Convolvulus oleifolius* Desr.**

Sheet 18 bottom: *Smyrnium creticum perfoliatum Jo. Bauh.*; location: *in Sicilia*. Taxon: ***Smyrnium perfoliatum* L.**

Sheet 19: *Sideritis montana salviae folio*; location: *ex Sicilia*. Unidentifiable.

Sheet 20 top: *Heliochrisum creticum*. Taxon: ***Helichrysum pendulum* (C. Presl) C. Presl**

Sheet 20 bottom: *Stoechas citrina tenuifolia*; location: *ex Sicilia*. Taxon: ***Helichrysum stoechas* (L.) Moench** or ***H. italicum* (Roth) G. Don**

Sheet 21 top: *Ranunculus Lusitanicus*; location: *in Sicilia*. Taxon: ***Ranunculus bullatus* L.**

Sheet 21 bottom: *Melilotus Italica follicu*[*li*]*s rotundis*; location: *in Sicilia*. Taxon: ***Melilotus italicus* (L.) Lam.**

Sheet 22 top: *Pistacium verum*; location: *Siculum*. Taxon: ***Pistacia vera* L.**

Sheet 22 middle: *Pistacii fructus*. Taxon: ***Pistacia vera* L.**

Sheet 22 bottom: *Acacia altera*; location: *Panormi Siculorum*. Taxon: ***Calicotome infesta* (C.Presl) Guss.**

Sheet 23 top: *Gnaphalium vescum*; location: *in Italia*. Taxon: ***Filago pygmaea* L.**?

Sheet 23 middle: *Lysimachia siliquosa rubra trifilla*; location: *in Gallia*. Taxon: ***Epilobium alpestre* (Jacq.) Krock.**

Sheet 23 bottom: *Arum Aegyptiacum*; location: *in Sicilia*. Taxon: ***Arum italicum* Mill.**

Sheet 24: *Pulegium minimum*; location: *Siculum*. Taxon: ***Mentha pulegium* L.**

Sheet 25 top: *Ferrum equinum capitatum*; location: *in Italia*. Taxon: ***Hippocrepis comosa* L.**

Sheet 25 bottom: *Geranium althaeoides*; location: *in Sicilia*. Taxon: ***Erodium malacoides* (L.) L’Hér.**?

Sheet 26 top: *Mandragora vera cum flore*; location: *Panormi*. Taxon: ***Mandragora officinarum* L.**

Sheet 26 bottom: *Seseli Massiliense folio foeniculi crassiore*; location: *in littore Liguriae*. Taxon: ***Seseli tortuosum* L.**

Sheet 27 top: *Androsaemum foetidum sive Tragodes*; location: *siculum*. Taxon: ***Hypericum hircinum* L. subsp. *majus* (Aiton) N.Robson**

Sheet 27 middle: *Hipericum tomentosum hispanicum*. Taxon: ***Hypericum tomentosum* L.**?

Sheet 27 bottom: *Geranium 7mum Dodonei*; location: *ubiq*[*uis*]. Taxon: ***Erodium cicutarium* (L.) L’Hér.**

Sheet 28 top: *Chamaegenista sagittalis*; location: *in Italia*. Taxon: ***Genista sagittalis* L.**

Sheet 28 bottom: *Genista Ilvensis*; location: *in Corsica*. Taxon: ***Genista umbellata* (L’Her.) Poir.** (a similar species *G. etnensis* (Raf.) DC. is a Corsican plant which Boccone might have confused here with.

Sheet 29 top: *Linaria latifolia trifilla*; location: *Sicula*. Taxon: ***Linaria triphylla* (L.) Mill.**

Sheet 29 middle: *Dorychnium monspeliense angustifolium*; location: *in Italia*. Taxon: ***Lotus dorycnium* L.**

Sheet 29 bottom: *Scabiosa arborea*; location: *in Creta et Sicilia*. Taxon: ***Lomelosia cretica* (L.) Greuter & Burdet**

Sheet 30 top: *Tarton raire sive Thymele*; location: *in Gallia*. Taxon: ***Thymelaea tarton-raira* (L.) All.**

Sheet 30 bottom: *Pimpinella spinosa*; location: *in Sicilia*. Taxon: ***Sarcopoterium spinosum* (L.) Spach** (=*Poterium spinosum* L.).

Sheet 31 left: *Ranunculus saxatilis Asphodeli radice*; location: *in Sicilia*. Taxon: ***Ranunculus paludosus* Poir.**

Sheet 31 right: *Nasturtium Asari foliis*; location: *in montibus Hetruriae*. Taxon: ***Cardamine asarifolia* L.**

Sheet 31 bottom: *Plantago laciniata Columnae*; location: *Sicilia Panormi*. Taxon: ***Plantago coronopus* L.**

Sheet 32 top: *Geranium tuberosum*; location: *prope Spoletum*. Taxon: ***Geranium tuberosum* L.**

Sheet 32 bottom: *Geranium insupra* [Lat.: “the above geranium”]. Taxon: ***Geranium tuberosum* L.**

Sheet 33 top: *Stoechas citrina brevioribus ligulis*; location: *in Sicilia*. Taxon: ***Staehelina dubia* L.**

Sheet 33 bottom: *Aster montanus tomentosus*; location: *in Italia*. Taxon: ***Inula montana* L.**

Sheet 34 top: *Smilax aspera sive Salsa parilla Italorum*; location: *in Sicilia*. Taxon: ***Smilax aspera* L.**

Sheet 34 bottom: *Ferrum equinum*; location: *Sferracavallo ex Italia*. Taxon: ***Hippocrepis comosa* L.**

Sheet 35 top: *Eryngium Planum*; location: *Siculum*. Taxon: ***Eryngium pusillum* L.**

Sheet 35 middle: *Thlaspi amarum*; location: *in Gallia*. Taxon: ***Iberis amara* L.**

Sheet 35 bottom: *Scabiosa montana candida*; location: *in Sabaudia*. Taxon: ***Cephalaria*** sp.

Sheet 36 top: *Sinapi agreste Columnae*; location: *in montibus Italiae*. Taxon: ***Chelidonium majus* L.**

Sheet 36 middle: *Bassica saxatilis Italica*; location: *in Corsica*. Taxon: ***Brassica insularis* Moris**

Sheet 36 bottom: *Clematis maritima laciniatis foliis*; location: *Neapoli*. Taxon: ***Clematis flammula* L.**

Sheet 37 top: *Filix maior ceterach foliis*; location: *America*. Taxon: ***Dryopteris filix-mas* (L.) Schott**

Sheet 37 middle: *Euphrasia sylvestris purpurea Dodonei*; location: *ex Italia*. Taxon: ***Odontites vulgaris* Moench**

Sheet 37 bottom: *Loto affinis siliquis circinnatis*; location: *ex Italia*. Taxon: ***Anthyllis circinnata* (L.) D.D. Sokoloff**

Sheet 38 top: *Alipum montis Ceti sive Thymelea capitulis scabiosae*; location: *in Sicilia et Corsica*. Taxon: ***Globularia alypum* L.**

Sheet 38 bottom: *Eringium capitulis psillii*. Taxon: ***Eryngium triquetrum* Vahl**

Sheet 39 top: *Chondrilla marina tuberosa*; location: *in littore Italico*. Taxon: ***Sonchus bulbosus* (L.) N. Kilian & Greuter**

Sheet 39 middle: *Leontopodium*; location: *in montibus Italiae*. Taxon: ***Leontopodium nivale* (Ten.) A. Huet ex Hand.-Mazz.** subsp. ***alpinum* (Cass.) Greuter**

Sheet 39 bottom: *Lotus siliquis ornitopodii*; location: *in Italia*. Taxon: ***Lotus ornithopodioides* L.**

Sheet 40 top: *Beta italica semine aculeato*; location: *Sicilia*. Taxon: ***Rumex spinosus* L.** (= *Emex spinosa* (L.) Campd.).

Sheet 40 bottom: *Crithmum siculum*; location: *Panormi*. Taxon: ***Seseli bocconei* Guss.**

Sheet 41 top: *Thymbra sicula Camphoratae foliis*; location: *Sicilia*. Taxon: ***Thymbra spicata* L.**

Sheet 41 bottom: *Asphodelus fistulosus*; location: *Sicilia*. Taxon: ***Asphodelus fistulosus* L.**

Sheet 42 top: *Gramen crucis Neiemelsalib*; location: *Sicula*. Taxon: ***Eleusine indica* (L.) Gaertn.**

Sheet 42 bottom: *Campanula Caetana*; location: *Neapolitana*. Taxon: ***Campanula fragilis* Cirillo**

Sheet 43 top: *Hypecoon corniculatum*; location: *ex Apulia*. Taxon: ***Hypecoum littorale* Wulfen**

Sheet 43 bottom: *Flos adonis flore luteo*; location: *ex Sicilia*. Taxon: ***Adonis vernalis* L.**

Sheet 44 top: *Helleborus niger legitimus Clusii*; location: *ex Corsica*. Taxon: ***Helleborus niger* L.**

Sheet 44 middle: *Rubia undulato folio montana*; location: *ex Sicilia*. Taxon: ***Rubia peregrina* L.**

Sheet 44 bottom: *Centaurea maior laciniatis foliis*; location: *ex America*. Taxon: ***Rhaponticoides centaurium* (L.) M.V. Agab. & Greuter**

Sheet 45: *Marrubium siculum pseudodictamni foliis*; location: *siculum*. Taxon: ***Marrubium vulgare* L.**

Sheet 45b top: *Filix saxatilis Ponae*; location: *in montibus Hetruriae*. Taxon: ***Cryptogramma crispa* (L.) R.Br.**

Sheet 45b bottom: *Veronica montana latifolia*; location: *in montibus Sabaudiae*. Taxon: ***Veronica urticifolia* Jacq.**

Sheet 46 left: *Ruscus*; location: *in Italia*. Taxon: ***Ruscus aculeatus* L.**

Sheet 46 right: *Thymelaea sive Tartonraira Gallorum*; location: *ex Corsica et Marsilia*. Taxon: ***Thymelaea tarton-raira* (L.) All.**

Sheet 46 bottom: *Chamaepythis tertia*; location: *Liburni*. Taxon: ***Cressa cretica* L.**

Sheet 47 left: *Heliotropium tricoccos*; location: *siculum*. Taxon: ***Chrozophora tinctoria* (L.) A. Juss.**

Sheet 47 right: *Mentha cattaria angustifolia*; location: *in montibus Sabaudiae*. Taxon: ***Nepeta nepetella* L.**

Sheet 47 bottom: *Rosa sinensis*; location: *ex India*. Taxon: ***Abutilon*** sp.

Sheet 48 top: *Drypis Theophrasti*; location: *in patria divi Benedicti Nursica*. Taxon: ***Drypis spinosa* L.**

Sheet 48 middle: *Cytisus Maranthae*; location: *in Sicilia et Neapoli*. Taxon: ***Medicago arborea* L.**

Sheet 48 bottom: *Nasturtium erectum*; location: *in Italia*. Taxon: ***Cardamine impatiens* L.**

Sheet 49 top: *Trifolium vesicarium*; location: *in Sicilia*. Taxon: ***Tripodion tetraphyllum* (L.) Fourr.**

Sheet 49 middle: *Dictamnus creticus*; location: *in Creta tantum*. Taxon: ***Origanum dictamnus* L.**

Sheet 49 bottom: *Calamintha montana prestantior*; location: *in Hetruria*. Taxon: ***Clinopodium grandiflorum* (L.) Kuntze**

Sheet 50 top: *Ammi verum*; location: *Italicum*. Taxon: ***Trachyspermum ammi* (L.) Sprague**

Sheet 50 middle: *Linum sylvestre latifolium*; location: *ex Liguria*. Taxon: ***Linum hirsutum* L.**

Sheet 50 bottom: *Saxifraga Foeniculi folio*; location: *in Moravia et in Sicilia*. Taxon: ***Ptychotis*** ***saxifraga*** **L.**

Sheet 51 top: *Marum Cortusi*; location: *in Corsica*. Taxon: ***Teucrium marum* L.**

Sheet 51 middle: *Gramen tremulum maius*; location: *in Italia*. Taxon: ***Briza maxima* L.**

Sheet 51 bottom: *Arenaria*; location: *ex Hollandia*. Taxon: ***Sagina nodosa* (L.) Fenzl**

Sheet 52 top: *Storax*; location: *Marsiliae*. Taxon: ***Styrax officinalis* L.**

Sheet 52 middle: *PseudoAsphodelus*; location: *in Corsica*. Taxon: ***Narthecium reverchonii* Čelak**

**Figure 1:**
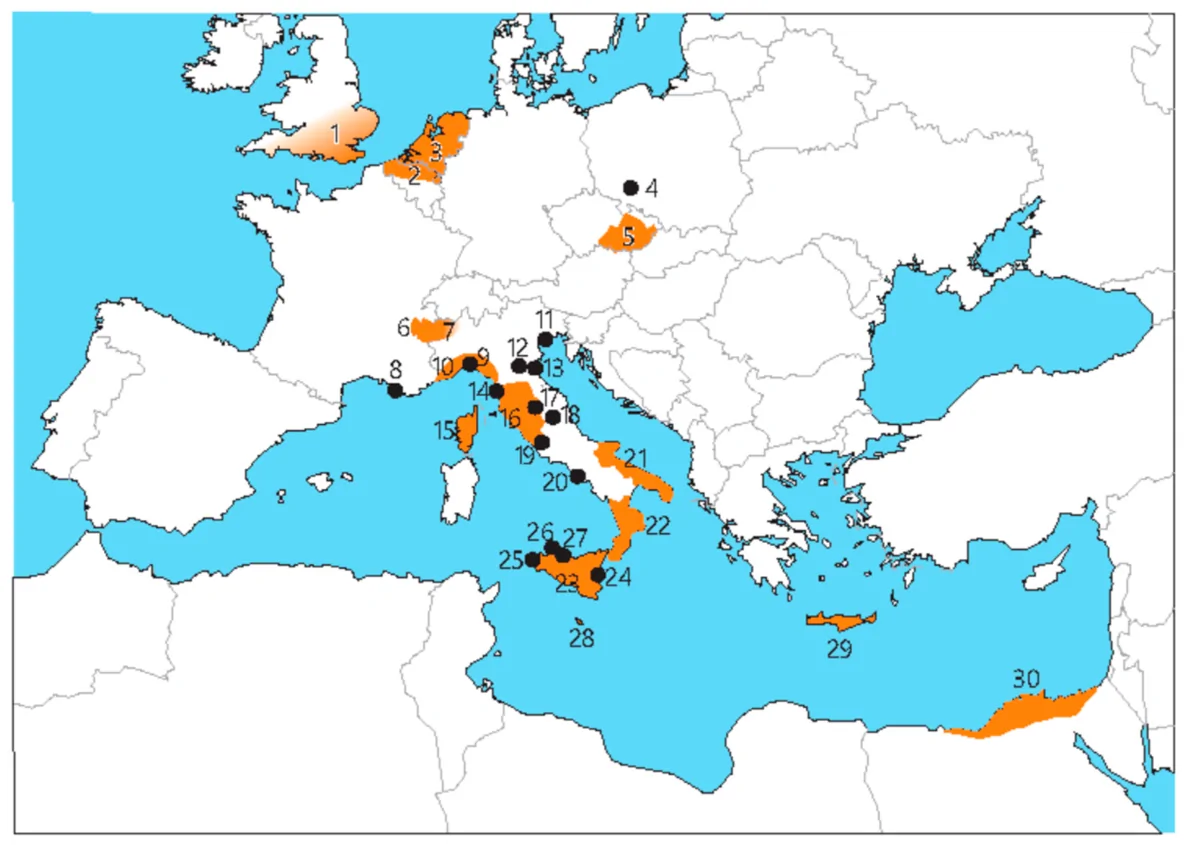
Towns (as black dots), regions and islands (orange areas) mentioned in the Boccone’s herbarium in Wrocław. Original *Latin* toponyms are given in brackets: 1—England (*Anglia*), 2—Flanders (*Flandria*), 3—The Netherlands (*Hollandia*), 4—Wrocław (*Wratislavia*), 5—Moravia (*Moravia*), 6—Savoy (*Sabaudia*), 7—Alps (*Alpi Hetruriae*), 8—Marseille (*Marsilia*), 9—Genoa (*Genua*), 10—Liguria (*Liguria*), 11—Venice Lido (*Lido Venetorum*), 12—Bologna (*Bononia*), 13—Ravenna (*Ravelna*), 14—Livorno (*Liburnum*), 15—Corsica (*Corsica*), 16—Etruria (*Hetruria*), 17—Spoleto (*Spoletum*), 18—Norcia (*Nursia*), 19—Rome (*Roma*), 20—Naples (*Neapolis*), 21—Apulia (*Apulia*), 22—Calabria (*Calabria*), 23—Sicily (*Sicilia*, adjective: *Siculus*), 24—Catania (*Catana*), 25—Trapani (*Drepanum*), 26—Sferracavallo (*Sferracavallo*), 27—Palermo (*Panormum*), 28—Malta (*Melita*), 29—Crete (*Creta*), 30—Egypt (*Aegyptus*).

Sheet 52 bottom: *PseudoAsphodelus spicatus luteus iridis folio*; location: *in Corsica*. Taxon: ***Narthecium reverchonii* Čelak**. See Figure 1.

Sheet 53 top: *Epimedium Dodonei*; location: *in Gallia*. Taxon: ***Epimedium alpinum* L.**

Sheet 53 bottom: *Anonis non spinosa flore coeruleo*; location: *in Italia*. Taxon: ***Ononis natrix* L.**

Sheet 54 top: *Gentiana cruciata*; location: *ubiq*[*ue*] *nascitur*. Taxon: ***Gentiana cruciata* L.**

Sheet 54 middle: *Gentiana asclepiadis folio*; location: *in Italia*. Taxon: ***Gentiana asclepiadea* L.**

Sheet 54 bottom: *Chondrilla rara Lobellii*; location: *ex Sicilia*. Taxon: ***Crupina crupinastrum* (Moris) Vis.**

Sheet 55 top: *Coronopus repens Ruellii*; location: *ubique in Italia*. Taxon: ***Lepidium coronopus* (L.) Al-Shehbaz**

Sheet 55 middle: *Alyssum Dioscoridis*; location: *in Italia*. Taxon: ***Fibigia clypeata* (L.) Medik.**

Sheet 55 bottom: *Alsine alpina angustifolia*; location: *in montibus Sabaudiae*. Taxon: ***Arenaria aggregata* (L.) Loisel.**

Sheet 56 top: *Onobrichis Italica tenuifolia*; location: *ex Italia*. Taxon: ***Hippocrepis scabra* DC.**?/***H. comosa* L.**?

Sheet 56 middle: *Nasturtium minimum saxatile*; location: *ex Liguria*. Taxon: ***Hornungia alpina* (L.) O.Appel**

Sheet 56 bottom: *Nasturtium Rhesedae foliis*; location: *ex Hetruria*. Taxon: ***Cardamine resedifolia* L.**

Sheet 57: *Lentiscus Peruanus sive Molle arbor Clusii*; location: *ex America*. Taxon: ***Schinus molle* L.**

Sheet 58: *Caryophyllus minimus montanus*; location: *Roma*. Taxon: ***Petrorhagia saxifraga* (L.) Link**

Sheet 59 top: *Euphrasia sicula vermiculata*; location: *ex Sicula*. Taxon: ***Euphrasia*** sp.?

Sheet 59 bottom: *Dryopteris*; location: *ex Sicula*. Taxon: ***Asplenium onopteris* L.**

Sheet 60 top: *Virga aurea hirsuta*; location: *Americana*. Taxon: ***Solidago canadensis* L.**

Sheet 60 bottom: *Geranium moschatum*; location: *ubiq*[*ue*] *et apud nos*. Taxon: ***Erodium moschatum* (L.) L’Hér.**

Sheet 61 top: *Bombax*; location: *in Melite*. Taxon: ***Gossypium*** sp.

Sheet 61 bottom: *Astragalus flore conglomerato* [*f*]*usco*; location: *in montibus Sabaudiae*. Taxon: ***Astragalus monspessulanus* L.**

Sheet 62: *Geranium siculum malvae folio rarissimum*. Taxon: ***Erodium malacoides* (L.) L’Hér.?**

Sheet 63 top: *Lonchitis maranthae*; location: *ex Corsica*. Taxon: ***Hemionitis marantae* (L.) Christenh.**

Sheet 63 bottom: *Geranium Chrysanthemi cretici foliis dissectis*; location: *in Apulia*. Taxon: ***Erodium ciconium* (L.) L’Hér.**

Sheet 64 top: *Polygonatum rectum Canadense*; location: *ex America*. Taxon: ***Polygonatum*** sp.

Sheet 64 bottom: *Paliurus sive Rhamnu*; location: *ex Sicilia*. Taxon: ***Paliurus spina-christi* Mill.**

Sheet 65 top: *Eruca saxatilis Italica*; location: *in montibus Etruriae*. Taxon: ***Cardamine parviflora* L.**

Sheet 65 bottom: *Pyrola*; location: *in montibus Sabaudiae*. Taxon: ***Orthilia secunda* (L.) House**

Sheet 66 top: *Cytisus rotundifolius*; location: *in Italia*. Taxon: ***Cytisophyllum sessilifolium* (L.) O.Lang**

Sheet 66 middle: *Coris matth*[*ioli*]; location: *in montibus Hetruriae*. Taxon: ***Hypericum coris* L.**

Sheet 66 bottom: *Helichrysu*[*m*] *silvestri affinis*; location: *in Sicilia*. Unidentifiable.

Sheet 67: *Trachelium latifolium*; location: *ex Flandria 1674 collecta â Rev. cum Bocconi*. Taxon: ***Campanula trachelium* L.**

Sheet 68 top: *Geranium myrrhidis folio*; location: *ubique in Italia*. Taxon: ***Erodium cicutarium* L’Hér.**

Sheet 68 bottom: *Thymaelea alpina acuminato folio*; location: *in Corsica*. Taxon: ***Daphne oleoides* Schreb.**?

Sheet 69 top: *Geranium Althaeae sinuato folio*; location: *ex Sicilia*. Taxon: ***Erodium chium* (L.) Willd.**

Sheet 69 middle: *Napellus*; location: *ubique in hortis invenitur*. Taxon: ***Aconitum napellus* L.**

Sheet 69 bottom: *Ononis non spinosa lutea minima Columnae*; location: *ex Italia*. Taxon: ***Ononis pusilla* L.**

Sheet 70 left: *Cotyledon pyramidale*; location: *ex Italia*. Taxon: ***Saxifraga callosa* Sm.**

Sheet 70 right: *Horminum salviae rotundo folio*; location: *ex Apulia*. Unidentifiable.

Sheet 70 bottom: *Rubia montana saxatilis*; location: *ex Sicilia*. Taxon: ***Rubia peregrina* L.**

Sheet 71 top: *Foenum graecum silvestre*; location: *ex Italia*. Taxon: ***Trigonella gladiata* Steven ex M.Bieb.**

Sheet 71 bottom: *Doronicum verum Columnae*; location: *Roma*. Taxon: ***Doronicum grandiflorum* Lam.**?/***D. pardalianches* L.**?

Sheet 72 top: *Valeriana Indica*; location: *ex Sicilia*. Taxon: ***Valeriana cornucopiae* L.**

Sheet 72 middle: *Circea montana minima Columnae*; location: *ex Italia*. Taxon: ***Circaea alpina* L.**

Sheet 72 bottom: *Sideritis Hyssopi folio*; location: *ex Italia*. Taxon: ***Sideritis hyssopifolia* L.**

Sheet 73 top: *Thalictrum montanum*; location: *ex Italia*. Taxon: ***Thalictrum minus* L.** subsp. ***minus***

Sheet 73 bottom: *Scabiosa gramineo folio*; location: *ex Sicilia*. Taxon: ***Lomelosia graminifolia* (L.) Greuter & Burdet**

Sheet 74: *Palmae Indicae species*; location: *ex India*. Taxon: ***Bactris hirta* Mart.**

Sheet 75: *Sideritis montana Lamii foliis*; location: *ex Hetruria*. Taxon: ***Ballota nigra* L.**

Sheet 76: *Panax Herculeum siculum Bocconi*; location: [*siculum*]. Taxon: ***Heracleum*** sp.

Sheet 77: *Panacis Herculei Folium*; location: *ex Sicilia*. Taxon: ***Heracleum*** sp.

Sheet 78 top: *Cynocrambe*; location: *ex Italia*. Taxon: ***Theligonum cynocrambe* L.**

Sheet 78 left: *Linaria sicula*; location: [*sicula*]. Unidentifiable.

Sheet 78 right: *Marum Cortusi*; location: *ex Corsica*. Taxon: ***Teucrium marum* L.**

Sheet 79 top: *Filix florida*; location: *ex Italia*. Taxon: ***Osmunda regalis* L.**

Sheet 79 bottom: *Trubulus terrestris*; location: *ex Sicula*. Taxon: ***Tribulus terrestris* L.**

Sheet 80 top: *Satureja spicata*; location: *ex Neapoli*. Taxon: ***Micromeria juliana* (L.) Benth. ex Rchb.**

Sheet 80 bottom: *Trifolium rubrum maculatum*; location: *in Calabria*. Taxon: ***Trifolium pratense* L.** subsp. ***semipurpureum* (Strobl) Pignatti**

Sheet 81 top: *Antillis*; location: *ex Melite*. Taxon: ***Camphorosma monspeliaca* L.**?

Sheet 81 bottom: *Hedysarum trifillum Canadense Cornuti*; location: *ex America*. Taxon: ***Desmodium canadense* (L.) DC.**

Sheet 82 top: *Aster myrthi folio*; location: *ex Italia*. Taxon: ***Buphthalmum salicifolium* L.**?

Sheet 82 middle: *Aster conyzoides umbellatus*; location: *in Hetruria*. Unidentifiable.

Sheet 82 bottom: *Phyllirea maior*; location: *ex Italia*. Taxon: ***Phillyrea latifolia* L.**

Sheet 83 top: *Teucrium Boeticum Clusii*; location: *ex Sicilia*. Taxon: ***Teucrium fruticans* L.**

Sheet 83 middle: *Hemionitis Matthioli*; location: *ex Sicilia*. Taxon: ***Asplenium sagittatum* (DC.) Bange**

Sheet 83 bottom: *Tithymalus spinosus*; location: *ex Hetruria*. Taxon: ***Euphorbia spinosa* L.**

Sheet 84: *Gramen aculeatum*; location: *ex Sicilia*. Taxon: ***Sporobolus aculeatus* (L.) P.M. Peterson**

Sheet 85 left: *Flos passionis hirsuto folio*; location: *Americana*. Taxon: ***Passiflora foetida* L.**

Sheet 85 right: *Coniza Alato caule cum flore*; location: *in Hetruria*. Taxon: ***Crepis* sp.?**

Sheet 86: *Coniza Alato caule*; location: *in Hetruria*. Unidentifiable., same as on sheet 85.

Sheet 87 top: *Phylliris ramosa latifolia*; location: *ex Sicilia*. Taxon: ***Pteris cretica* L.**

Sheet 87 bottom: *Phylliris ramosa angustifolia*; location: *ex Corsica*. Taxon: ***Pteris vittata* L.**

Sheet 88 top: *Adianthum Canadense Cornuti*; location: *ex America*. Taxon: ***Adiantum pedatum* L.**

Sheet 88 middle: *Ranunculus Alopecuroides Ajugae foliis*; location: *Massiliae*. Taxon: ***Ranunculus falcatus* L.**

Sheet 88 bottom: *Medica marina*; location: *in littore Italico*. Taxon: ***Medicago marina* L.**

Sheet 89 top: *Cistus mas*; location: *in Italia*. Taxon: ***Cistus albidus* L.**

Sheet 89 middle: *Cistus foemina*; location: *in Italia*. Taxon: ***Cistus salviifolius* L.**

Sheet 89 bottom: *Moly pesariense Ponae*; location: *in Italia*. Taxon: ***Allium moly* L.**

Sheet 90: *Sebesten*; location: *ex insula Melite*. Taxon: ***Cordia myxa* L.**

Sheet 91 left: *Polygala sive flos ambarualis Dodonae*; location: *ubique locorum*. Taxon: ***Polygala vulgaris* L.**

Sheet 91 right: *Rhus myrti folio*; location: *Genua*. Taxon: ***Coriaria myrtifolia* L.**

Sheet 92 top: *Stoechas Arabica*; location: *in Italia*. Taxon: ***Lavandula stoechas* L.**

Sheet 92 middle: *Linaria alba Lobell.*; location: *in Sicilia*. Taxon: ***Linaria chalepensis* (L.) Mill.**

Sheet 92 bottom: *Laurus tinus*; location: *in Italia*. Taxon: ***Viburnum tinus* L.**

Sheet 93 top: *Limonium maritimum Bellidis minoris folio*; location: *Melite*. Taxon: ***Limonium*** sp.

Sheet 93 middle: *Ficus Indica sive Opuntia*; location: *in Sicilia*. Taxon: ***Opuntia ficus-indica* (L.) Mill.**

Sheet 93 bottom: *Strutium antiquorum sive Herba lanaria Imperati*; location: *in Sicilia*. Taxon: ***Gypsophila struthium* L.**

Sheet 94 top: *Limonium bellidis folio acuto*; location: *in Italia Ravelnae*. Taxon: ***Limonium narbonense* Mill.**

Sheet 94 bottom: *Genista Italiae tinctorum*; location: *ubique locor*[*um*]. Taxon: ***Genista tinctoria* L.**

Sheet 95 top: *Panax Asclepias*; location: *ex Sicilia*. Taxon: ***Ferulago galbanifera* (Mill.) W.D.J. Koch**?

Sheet 95 middle: *Thlaspi folio Amaraci*; location: *in montibus Hetruria*[*e*]*, nascitur etiam apud nos Wratislaviae*. Taxon: ***Odontarrhena argentea* (All.) Ledeb.**

Sheet 95 bottom: *Thlaspi biscutatum Hieracii folio*; location: *ex Insula Melita*. Taxon: ***Biscutella laevigata* L.**

Sheet 96 top: *Coris monspeliensis*; location: *in montibus Hetruriae*. Taxon: ***Coris monspeliensis* L.** subsp. ***monspeliensis***

Sheet 96 middle: *Lychnis viscosa*; location: *in Italia*. Taxon: ***Silene* cf. *otites* (L.) Wibel**

Sheet 96 bottom: *Lychnis viscosa*. Taxon: ***Viscaria vulgaris* Bernh.?** or the same as on sheet 96 (middle) again.

Sheet 97 top: *Chamaedrys spinosa*; location: *ex Sicilia*. Taxon: ***Teucrium spinosum* L.**

Sheet 97 bottom: *Musa serapionis*; location: *ex Aegÿpto et Sicilia*. Taxon: ***Musa* ×*paradisiaca* L.**

Sheet 98 top left: *Spina solstitialis*; location: *ex Sicilia*. Taxon: ***Centaurea solstitialis* L.**

Sheet 98 bottom left: *Umbilicus Veneris matth.*; location: *in Italia*. Taxon: ***Umbilicus*** sp.

Sheet 98 right: *Atractylis pyramidalis sicula*; location: *ex Italia*. Taxon: ***Phonus lanatus* (L.) Hill**?/***Scolymus hispanicus* L.**?/***Atractylis cancellata* L.**?

Sheet 99 top left: *Cistus plantaginis folio cum flore*; location: *in insula Corsica*. Taxon: ***Tuberaria lignosa* (Sweet) Samp.**

Sheet 99 top right: *Thymum creticum verum*; location: *ex Sicilia*. Taxon: ***Thymbra capitata* (L.) Cav.**

Sheet 99 middle: *Convolvulus spicaefolius*; location: *ex Melite*. Taxon: ***Convolvulus cantabrica* L.**

Sheet 99 bottom right: *Cistus plantaginis folio*. Taxon: ***Tuberaria lignosa* (Sweet) Samp.**

Sheet 100 top: *Chamaecistus siliquis nutantibus*; location: *ex Sicilia*. Taxon: ***Helianthemum aegyptiacum* (L.) Mill.**

Sheet 100 middle: *Scabiosa nana argentea*; location: *ex Sicilia et Creta*. Unidentifiable.

Sheet 100 bottom: *Scabiosa arborea cretica*. Taxon: ***Lomelosia cretica* (L.) Greuter et Burdet**

Sheet 101 top: *Cistus annuus flore guttato*; location: *ex Sicilia et ex Hetruria*. Taxon: ***Tuberaria guttata* (L.) Fourr.**

Sheet 101 middle: *Cistus ut supra* [Lat.: “as above”]. Taxon: ***Tuberaria guttata* (L.) Fourr.**

Sheet 101 bottom: *Chamaecistus thymifolio*; location: *ex Sicilia*. Taxon: ***Fumana thymifolia* (L.) Spach ex Webb**

Sheet 102 top: *Convolvulus argenteus pentaphylli*; location: *ex Sicilia*. Taxon: ***Momordica charantia* L.**?

Sheet 102 bottom: *Horminum betonicae folio*; location: *ex Anglia*. Taxon: ***Salvia verbenaca* L.**

Sheet 103 top: *Erica montana flore rubello*; location: *ex Sicilia*. Taxon: ***Erica sicula* Guss.**

Sheet 103 bottom right: *Horminum odoratum verbenae folio*; location: *ex Roma*. Taxon: ***Salvia verbenaca* L.**

Sheet 103 bottom left: *Horminum idem* [Lat.: “the same”]. Taxon: ***Salvia verbenaca* L.**

Sheet 104 top: *Dorychnium argenteum*; location: *ex Sicilia et Creta*. Taxon: ***Convolvulus cneorum* L.**

Sheet 104 bottom: *Chrysanthemum hispanicum valentinum*; location: *ex Sicilia*. Taxon: ***Anacyclus clavatus* (Desf.) Pers.**

Sheet 105 top: *Atriplex sylvestris*; location: *ex Sicilia*. Unidentifiable.

Sheet 105 middle: *Bellis alpina Tanaceti dissectis foliis*; location: *ex Sicilia*. Taxon: ***Cota austriaca* (Jacq.) Sch.-Bip.**

Sheet 105 bottom: *Alsine lotoides Bocconi*; location: *ex Sicilia*. Taxon: ***Glinus lotoides* L.**

Sheet 106 top: *Myrthus Italica fructu albo*; location: *ex Sicilia*. Taxon: ***Myrtus communis* L.**

Sheet 106 bottom: *Borago minima aquatica*; location: *ex Creta et Insula Corsica*. Unidentifiable.

Sheet 107 top: *Esula rara*; location: *ex Li*[*d*]*o Venetorum Insula*. Unidentifiable.

Sheet 107 middle: *Linum silvestre latifolium*; location: *in Apulia*. Taxon: ***Linum maritimum* L.**?/***L. flavum* L.**?

Sheet 107 bottom: *Ceratonia sive Carrub*. Taxon: ***Ceratonia siliqua* L.**

Sheet 108: *Muscus sive Corallina nigra ramosa*; location: *in Oceano*. Unidentifiable.

Sheet 109 top: *Porus cervinus Imperati*; location: *in Mari Siculo*. Unidentifiable.

Sheet 109 bottom: *Mirica marina*; location: *in Mari Siculo*. Unidentifiable.

Sheet 110 top: *Pinna marina fruticosa*; location: *in Mari Siculo*. Unidentifiable.

Sheet 110 middle: *Fucus marinus Rosmarini dentatis foliis*; location: *in Oceano*. Taxon: ***Fucus serratus* L.**

Sheet 110 bottom: *Erica marina*; location: *in Mari Siculo*. Unidentifiable.

Sheet 111 top: *Spongia ramosa marina*, [Germ.:] *Meer Schwamm mit Ohre*. Unidentifiable.

Sheet 111 bottom: *Alga intybacea*, [Germ.:] *Meer Graß…* [unreadable]. Unidentifiable.

Sheet 112: [blank page].

Sheet 113: *Fucus arboreus polyschides*; location: *in Oceano*. Taxon: ***Saccorhiza polyschides* (Lightf.) Batt.**

Sheet 114 top: *Favago sive Vesicaria marina*; location: *in Oceano*. Unidentifiable.

Sheet 114 middle: *Tripolium Dodonaei*; location: *prope Liburnum*. Taxon: ***Tripolium pannonicum* (Jacq.) Dobrocz.**

Sheet 114 bottom: *Absinthium marinum*; location: *in littore Italico*. Taxon: ***Artemisia arborescens* L.**

Sheet 115 top: *Rhamnus pentaphylla sicula Bocconi*; location: *prope Panormum*. Taxon: ***Searsia pentaphylla* (Jacq.) F.A. Barkley ex Moffett**

Sheet 115 bottom: *Fucus angustifolius ligulas referens*; location: *in Oceano*. Taxon: ***Zostera*** sp.?

Sheet 116 top: *Jacobaea marina*; location: *in littore Maris Italici*. Taxon: ***Jacobaea maritima* (L.) Pelser & Meijden**

Sheet 116 middle: *Laurus Alexandrina Myrthi folio*; location: *ex India*. Taxon: ***Danaë racemosa* (L.) Moench**

Sheet 116 bottom: *Laurus Alexandrina ut sup*[*ra*] [Lat. “as above”]. Taxon: ***Danaë racemosa* (L.) Moench**

Sheet 117 top: *Rubia maritima*; location: *in lit*[*t*]*ore Siculo et Veneto*. Taxon: ***Crucianella maritima* L.**

Sheet 117 bottom: *Gnaphalium maritimum*; location: *in littore Siculo et Veneto*. Taxon: ***Achillea maritima* (L.) Ehrend. & Y.P. Guo**

Sheet 118 top: *Spongia ramosa arborea*; location: *in Oceano*. Unidentifiable.

Sheet 118 middle: *Fucus maritimus Abrotonoides*; location: *in Oceano*. Unidentifiable.

Sheet 118 bottom: *Fucus foliculaceus maritimus*; location: *in Oceano*. Unidentifiable.

Sheet 119 top: *Fucus maritimus minor triphydo folio*; location: *in Oceano*. Unidentifiable.

Sheet 119 middle: *Opuntia maritima*; location: *in Mari siculo*. Unidentifiable.

Sheet 119 bottom: *Alga rubra angustifolia maritima*; location: *in Oceano et Siculo Mari*. Unidentifiable.

Sheet 120 top left: *Leucoium maritimum*; location: *in littore siculo*. Taxon: ***Matthiola tricuspidata* (L.) W.T. Aiton**

Sheet 120 top right: *Leucoium maritimum crucigaerum*. Taxon: ***Matthiola tricuspidata* (L.) W.T. Aiton**

Sheet 120 middle: *Halimi flor*[*es*]; location: *in littore Italico et Siculo*. Taxon: ***Atriplex halimus* L.**

Sheet 120 bottom left: *Portulaca marina sive Halimus minor*. Taxon: ***Halimione portulacoides* (L.) Aellen**?

Sheet 120 bottom right: *Polygonum maritimum minimum Thymi folio*; location: *in littore Siculo*. Taxon: ***Frankenia laevis* L.**

Sheet 121 top: *Pastinaca marina*; location: *in littore Italico et Siculo*. Taxon: ***Echinophora spinosa* L.**

Sheet 121 bottom: *Eruca marina sive Kachile Serapionis*; location: *in littore Siculo et Veneto*. Taxon: ***Cakile maritima* Scop.**

Sheet 122 top: *Polygonum argenteum maritimum*; location: *Siculum*. Taxon: ***Paronychia argentea* Lam.**

Sheet 122 middle: *Kali floridum semine cochleato Bocconi*; location: *Siculum*. Taxon: ***Salsola oppositifolia* Desf.**

Sheet 122 bottom: *Solanum somniferum antiquorum*; location: *in Creta et in Sicilia*. Taxon: ***Atropa belladonna* L.**

Sheet 123 top: *Fucus maritimus Auriculae iudae facie*; location: *in Mari Siculo et Ligurico*. Unidentifiable.

Sheet 123 bottom: *Quercus marina super saxum donata*; location: *in Oceano*. Taxon: ***Fucus serratus* L.**

Sheet 124 top: *Quercus maritima bullata*; location: *in Oceano*. Taxon: ***Fucus vesiculosus* L.**

Sheet 124 bottom left: *Lichen maritima*; location: *in Mari Siculo et Ligurico*. Unidentifiable.

Sheet 124 bottom right: *Uva marina*; location: *in Mari Siculo*. Taxon: ***Caulerpa racemosa* (Forsskål) J. Agardh**

Sheet 125: *Quercus marina* [Germ.:] *Meereiche*; location: *ex Mari Siculo*. Taxon: ***Fucus vesiculosus* L.**

Sheet 126: *Fucus longissimo latissimo crassoque folio maritimus, Fucus Arboreus Polychides*. *Bauhini*. [Germ.:] *Meer bäum…* [illegible]; location: *in Oceano*. Taxon: ***Saccharina latissima* (L.) C.E. Lane, C. Mayes, Druehl & G.W. Saunders.**

Sheet 127 top: *Linaria triphilla latifolia*; location: *Siculae*. Taxon: ***Linaria triphylla* (L.) Mill.**

Sheet 127 bottom: *Veronica alpina Melissae folio*; location: *in Alpibus Hetruriae et Sabaudiae*. Taxon: ***Veronica teucrium* L.**

Sheet 128 top: *Muscus maritimus lucidus*; location: *in Oceano*. Unidentifiable.

Sheet 128 bottom: *Conferva maritima*; location: *in Mari Siculo*. Unidentifiable.

Sheet 129: *Antipates coralloides*; location: *ex Sicilia*. Taxon: ***Alcyonium coralloides* (Pallas 1766)**, a soft-coral.

Sheet 130 top: *Corallina frutescens crocea*; location: *in Mari Siculo*. Unidentifiable.

Sheet 130 bottom: *Fucus maritimus vermiculatus viridis*; location: *in Mari Melitensi*. Unidentifiable.

Sheet 131 top: *Ambrosia Matthioli*; location: *in littore Siculo et Ligustico* [correctly: *Ligurico*]. Taxon: ***Ambrosia maritima* L.**

Sheet 131 bottom: *Soldanella sive Brassica marina*; location: *in littore siculo et ligurico*. Taxon: ***Calystegia soldanella* (L.) Roem. & Schult.**

Sheet 132 top: *Kali geniculatum*; location: *Liburni et Panormi*. Taxon: ***Salicornia europaea* L.**

Sheet 132 bottom: *Corallina palmata arborea*; location: *in Mari Siculo Drepani*. Unidentifiable.

Sheet 133 top: *Quercus marina repens*; location: *in Oceano*. Unidentifiable.

Sheet 133 bottom left: *Quercus marina multifido folio*; location: *in Oceano*. Unidentifiable.

Sheet 133 bottom right: *Quercus marina crustosa*; location: *in Oceano*. Unidentifiable.

Sheet 134: *Fucus maritimus tuberculis paucissimis*; location: *in Oceano*. Unidentifiable.

Sheet 135: *Trifolium procumbens capitulo scabiosae*; location: *Siculum*. Taxon: ***Medicago polymorpha* L.?**

Sheet 136: *Androsemum Campoclarense fabii Columnae*; location: *ubiq*[*ue*] *locor*[*um*] *nascitur*. Taxon: ***Hypericum perforatum* L.**

Sheet 137 top: *Anonis non spinosa flore subalbido*; location: *Sicula*. Taxon: ***Ononis natrix* L.**

Sheet 137 bottom: *Tithymalus Helioscopius maritimus*; location: *Siculus*. Taxon: ***Euphorbia helioscopia* L.**

Sheet 138 top: *Thlaspi creticum*; location: *in Italia etiam nascitur* [Lat. “…grows too”]. Taxon: ***Noccaea caerulescens* (J. Presl & C. Presl) F.K. Mey.**

Sheet 138 bottom: *Alni folio turbinato fructu*; location: *ex India*. Taxon: ***Heliotropium indicum* L.**

Sheet 139 top: *Cynoglossum incanum creticum*; location: *in Sicilia etiam invenitur* [Lat. “…is also found”]. Taxon: ***Cynoglossum creticum* L.**

Sheet 139 bottom: *Geranium coriandri folio*; location: *ex Sicilia*. Taxon: ***Pelargonium myrrhifolium* (L.) L’Hér. var. *coriandrifolium* (L.) Harv.**

Sheet 140 left: *Jacea nigra angusto integro folio*; location: *ubiq*[*ue*] *locor*[*um*] *nascitur*. Taxon: ***Centaurea jacea* L.** subsp. ***jacea***

Sheet 140 right: *Valeriana minor*; location: *ubique nascitur*. Taxon: ***Valeriana officinalis* L.**

Sheet 141 left: *Bellis ramosa umbellifera Cornuti*; location: *Indica*. Taxon: ***Erigeron annuus* (L.) Desf.**

Sheet 141 right: *Amaranthus spicatus Siculus*; location: *prope Catanam et al. ibi*. Taxon: ***Achyranthes aspera* L. var. *sicula* L.**

Sheet 142 top: *Sanamunda*; location: *in Sicilia prope Panormum*. Taxon: ***Thymelaea hirsuta* (L.) Endl.**

Sheet 142 bottom: *Carthamus sive Cnicus caeruleo flore*; location: *in Sicilia prope Panormum*. Taxon: ***Carthamus caeruleus* L.**

## 3. Discussion

### 3.1. Plant Locations vs. the Origin of Exotic Species

Locations of plants are sometimes parts of their polynomial names, e.g., *convolvulus Siculus*… (Latin: Sicilian bindweed), but most of them were written on the labels as a separate addition. We have reduced them all to modern countries, islands, oceans, seas and continents (for original and interpreted place names see the caption of Figure 1).

The frequency of locations of plants is as follows:

- Mediterranean area: 240 specimens (90.9% of the herbarium), including:
  ○ Sicily: 96 specimens (40% of Mediterranean plants);
  ○ Continental Italy: 93 specimens;
  ○ Ocean (coasts of northwestern Africa?): 17 specimens;
  ○ Corsica: 12 specimens;
  ○ Continental France: seven specimens;
  ○ Malta: seven specimens;
  ○ Crete: five specimens;
  ○ Egypt: two specimens;
  ○ Africa: one specimen.
- West Europe: four specimens:
  ○ England: two specimens;
  ○ Belgium: one specimen;
  ○ The Netherlands: one specimen.
- Central Europe: two specimens (mentioned as such):
  ○ Czech Republic (Moravia province): one specimen;
  ○ Poland (Wrocław city, cultivated): one specimen.
- America: 11 specimens.
- India: seven specimens.

Plants from “America” or “India” make 6.8% of this collection.

This geographical range corresponds with the title of Boccone’s taxonomical and medicinal study “Museum of Rare Plants of Sicily, Malta, Corsica, Italy, Piedmont and Germany” printed in 1697 [26].

Boccone was especially interested in rare or curious plants. He usually entitled his herbaria this way (e.g., his 1678 herbarium stored in Genova, see [27] (p. 441); for the one stored in Paris (see [28], p. 215). He also issued his 1697 catalogue under the title *Museo di piante rare…* [26]). Whether a plant is rare or interesting is a matter of subjective opinion, but he must certainly have enjoyed encountering exotic species. In order to encounter rare specimens, he not only went on field trips, but also visited botanical gardens [1] (p. 378). For exotic plants, he recorded their geographical origin rather than the location of the garden:

- ***Styrax officinalis* L.** (native to the eastern Mediterranean area and the Middle East), was labelled “Marsilliae” (sheet 52).
- Banana (***Musa* × *paradisiaca* L.**) originates from the Indo-Malaysian subkingdom [29] but was labelled “Egypt”—bananas have been cultivated in Africa since the Middle Ages [30].
- ***Bactris hirta* Mart.**, native to Brazil, was labelled “India”.

These localities are examples of the general biogeographical knowledge of his times.

By noting the country of origin of the plant, Boccone seems to have emphasised its exotic origins, thereby making his herbarium more appealing.

Some plant locations are incorrect. A member of the Apiaceae family, *Ptychotis saxifraga* (sheet No. 50), was labelled “in Moravia et in Sicilia” (in Moravia and Sicily), but it is an Italian and West-European plant, and Boccone might have confused *Ptychotis saxifraga* with a very similar *Pimpinella saxifraga* L. (an apiaceous plant common in Central Europe—[31]). Ground-level leaves of both these species are pinnate, leaflets are ovate with dentate margin, and both these species grow on rocks, so he may have treated *Pt. saxifraga* and *Pimp. saxifraga* as one.

### 3.2. Rare Wild Plants

**Figure 2:**
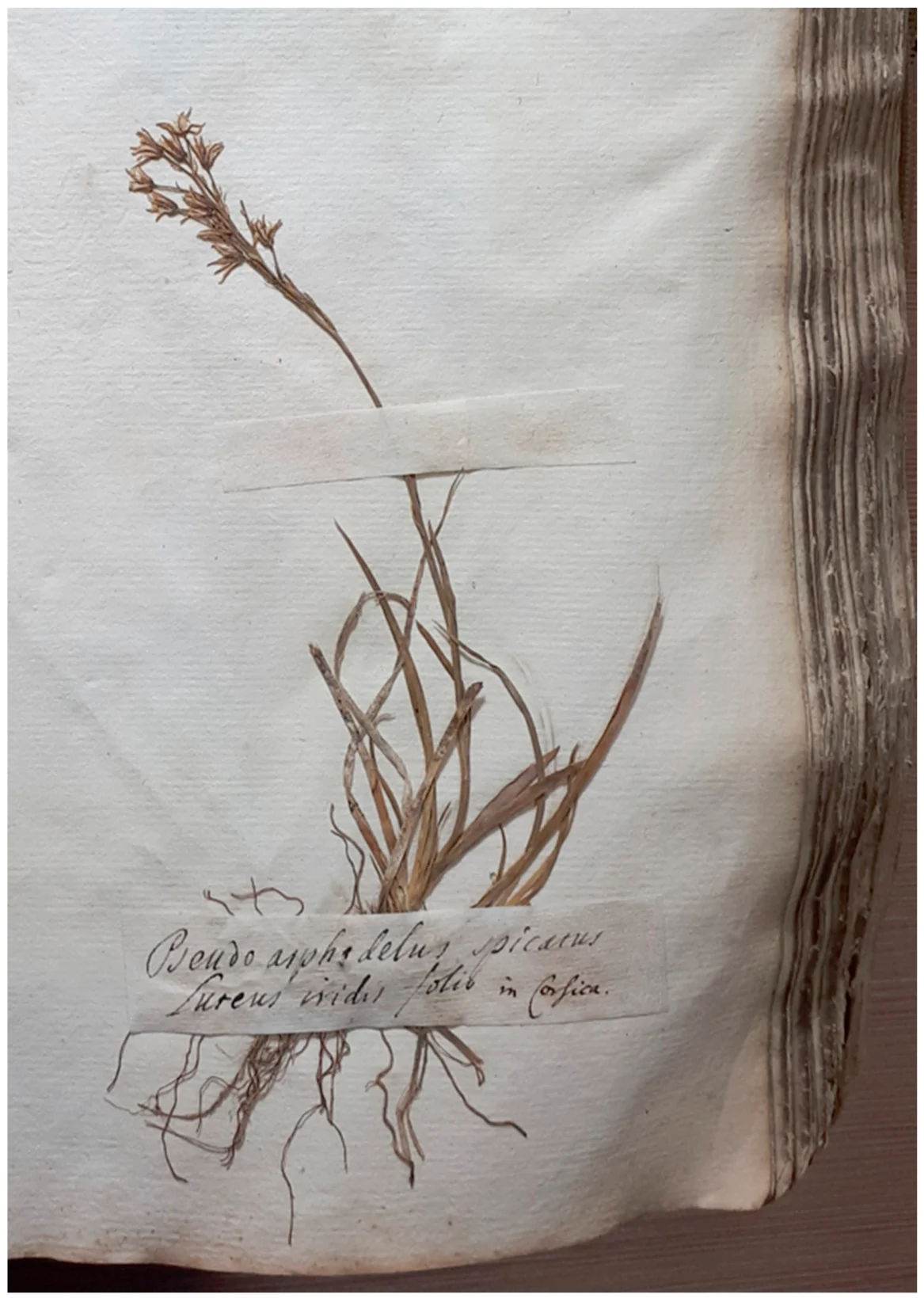
A specimen of *Narthecium reverchonii* Čelak, a species endemic to Corsica, from sheet 52 of Paolo Boccone’s herbarium kept in Wrocław (Poland). A photograph by Magdalena Grenda-Kurmanow.

Researchers studying Boccone’s scientific legacy agree that he collected plants for specific purposes, selecting them according to his interests. His main motive for creating herbaria was to search for rare species (see [31]). The contents of Boccone’s Wrocław collection seem to fully confirm this: indeed, it contains plants that are infrequent in the regions where they were found. Definitely, the most unique European plant in his collection is ***Narthecium reverchonii* Čelak** on sheet 52 (Figure 2), which is endemic to the mountains of Corsica. In contemporary times, the distribution of this species extends from the Cinto massif (Cintu) to the Bavella massif, where it is grows at altitudes ranging from 800 to 2200 metres above sea level [32].

### 3.3. Medicinal and Economic Exotic Plants

An additional motive of Boccone’s explorations was the search of rare plants grown in gardens: he visited apothecary gardens, ready to endure many hardships and inconveniences [1] (p. 378). However, the list of medicinal or other useful plants from gardens in the herbarium is very short and limited to rare exotics, grown in gardens at the time. The medicinal exotic plants known and used in Boccone’s time and present in his herbarium are listed below:

***Gossypium arboreum* L.** (sheets No. 3 and 61), a plant native to India [33], was a rare fibre-yielding plant in Bocone’s time, found by him as grown in Malta.

***Sapindus saponaria* L.** (sheet 8), labelled as “India” but native to Brazil [33], a source of saponins, valued as a surrogate of soap.

***Abutilon* sp.** (sheet No. 47), labelled as “India”, is a pantropical genus [33]. Species from this and allied genera have been used medicinally since Antiquity.

***Opopanax chironum* (L.) W. D. J. Koch** (labelled as such on sheet 98), but the plant specimen is in fact ***Ferulago galbanifera* (Mill.) W.D.J. Koch.** It is Boccone’s or a gardener’s mistake in identification. *O*. *chironum* was valued as an alleged “cure-all” remedy. *Ferulago* spp. yielded exudations (from incised roots) of medicinal importance.

Exotic medicinal plants of the Boccone’s time are only these four out of 134 specimens. Therefore, *materia medica* (medicinal plants) seem to not be the main motivation behind Boccone’s visits in gardens.

***Gypsophila struthium* Loefl.** (as Sicilian plant, sheet 93) is native to the Iberian Peninsula and Morocco [33] but since Antiquity it has been widely used as a plant for washing clothes (rich in saponins which produce foam as soap). Hence it was called in Latin *herba lanaria*—“wool herb” [34]. It could be cultivated elsewhere as an economic plant.

### 3.4. Ornamentals Which Later Became Medicinal Plants

It is worth noting that Boccone gathered other exotic plants, which later became famous as sources of herbal medicine. Our query in the pharmacological literature from the years 1574–1900 has established that: ***Adiantum pedatum* L.** (sheet 88) was recognised as medicinal in 1787 [35]; ***Schinus molle* L.** (sheet 57) was mentioned in 1574 [36] but wider described pharmacologically in the second half of the 18th century [37]; ***Passiflora foetida* L.** (sheet 85), of tropical and subtropical America [33], has been used medicinally since about 1758 [38]; ***Podophyllum peltatum* L.** (sheet 4) came to the attention of pharmacology in 1865 [39]; and ***Solidago canadensis* L.**, native to North America [33], was probably mentioned among uncertain species from the genus *Solidago* and proposed as medicinal in 1787 [35] (p. 123), but it is a modern medicinal species [40]. ***Desmodium canadense* (L.) DC.** (sheet No. 81) of North America [33] became medicinal in the first half of the 18th century [41] and better known since 1795 [42]. ***Athamanta cretensis* L.** (sheet 5, labelled as from Sicily) is not a Sicilian species. It became medicinal in the second half of the 18th century [43].

In Boccone’s time, these seven species must have been treated at most as exotic ornamental plants.

### 3.5. Was Boccone Involved in a Study of Herbal Medicinal Materials?

Boccone’s printed publications aimed to describe rare or curious (for him) plant species (most of them encountered for the first time) and to provide their iconography. His 1674 work entitled *Icones et descriptiones rariorum plantarum* [44] is an example of this. The only digression on pharmacology is found in the description of *Cynomorium coccineum* L., a well-known and unusual herbal remedy from Malta. Boccone knew and described medicinal applications of this plant.

Only in his final printed book *Museo di piante rare…* from 1697, Boccone presented medicinal topics, but he only collected and edited letters from medical practitioners who reported their floristic findings or tested certain plants as potential medicinal herbal materials [26]. Today this process is known as “seeking candidates for medicinal plants”. In the introduction, Boccone expressed his excitement about discovering medicinal uses for plants that had not been used before. Such results were presented in the first two chapters which were entitled *Osservazione*… (Italian for “Observation”, a term usually announcing a new scientific discovery). However, these were not his own results. Chapter 1 presented two plants: *thlaspi leucoji folio latifolium platycarpos, Siculum, semper virens* (later called *thlaspi latifolium platycarpon leucoii folio*, and identified by [28] (p. 221) as *Iberis semperflorens* L.), and *leucoium maritimum*, *crucigerum del Camerario*, present on sheet 120 of the Wrocław herbarium and identified by us as *Matthiola tricuspidata* (L.) W.T. Aiton. On p. 5, it says that, based on its similarity in taste, smell, and leaf thickness to the *Cochlearia* plant, as well as on the pungency, *M. tricuspidata*_has been ranked as possessing antiscorbutic properties. This is an example of the typical method used to identify new medicinal plants at that time. The second chapter dealt with a certain medicinal plant or herbal material, and the third one about compound medicines for *phtisis* (tuberculosis).

All the other observations in this book are floristic records from many parts of Europe (Germany, the Alps, Hungary, Sicily, Rome…), some of which focus on strongly scented odours (which might indicate their possible medicinal value). The polynomials mentioned in these records are, at most, similar to those used in Boccone’s herbarium (e.g., *Lysimachia siliquosa purpurea Alpina trifoliata* on p. 22 vs. *Lysimachia siliquosa rubra trifilla* on sheet 23).

In one place, a chapter about *chrysanthemum exoticum, incano cinerariae folio* (*Tanacetum cinerariifolium* (Trevir.) Sch.Bip.), Boccone added the following remark: “Today [this plant] has been lost, and I had it drawn from a flower and from the dried leaves that I found glued in my herbarium. On my last journey, I found it in great abundance on the Dalmatian Islands in the year 1694.” [26] (p. 23). His digression belongs to the field that would later be called pharmaceutical botany.

Only one medicinal plant, *Matthiola tricuspidata*, is found in both the Wrocław herbarium and the book in question. Therefore, this is merely a coincidence. The ratio of botanical to medical content in this book suggests that Boccone was much more interested in botany than medicine. Consequently, one should not expect to find his discoveries regarding the pharmacology of plant materials elsewhere.

### 3.6. Taxonomy and Difficulties in Identification

There are 313 specimens in the herbarium (including plants and marine organisms) mounted on 142 pages. No one genus is predominant in this collection; there are six *Hypericum* specimens, and four specimens each of the genera *Cardamine*, *Convolvulus*, *Erodium*, *Fucus*, and *Teucrium*. So, the pattern of this collection is variety rather than taxonomical specialisation.

Out of 313 specimens, 36 specimens (11, 5%) were unidentified by us. We have not identified 11 terrestrial plants due to the fact that they were collected in small fragments lacking generative organs or other diagnostic parts.

*Athamanta cretensis* (sheet 5, labelled as from Pallermo) and *Adonis vernalis* (sheet 47, labelled as from Sicily), are not native in Sicily [33]. They might have been gathered from a garden, as ornamental plants.

*Stahelina dubia* (sheet 27), *Ptychotis saxifraga* (sheet 50) and *Lomelosia graminifolia* (sheet 73), all labelled as from Sicily, are not Sicilian plants either. The place of origin might be confused by Boccone.

The herbarium also contains marine organisms that currently belong to the animal kingdom, such as algae, corals and sponges. We left 25 marine specimens unidentified. They require algological or zoological identification involving destructive sampling which is normally forbidden for historical and legally protected objects.

In past centuries, marine organisms were confused with plants or simply classified as plants due to their shape and sedentary lifestyle [1], and how they offered another source of *materia medica*, that is, medicinal raw materials. It is therefore no surprise that Boccone took an interest in them as a physician.

## 4. Materials and Methods

Plant identification was done in two ways: Plant specimens were identified taxonomically by botanists. The handwritten polynomial names of the plants, written on the herbarium labels, were carefully transcribed and searched in the historical floristic literature dating back to the 17th century. Many of Boccone’s polynomials have already been resolved in historical studies on his other herbaria, which became a clue for us. For this purpose, we used 19th-century studies by [27,28], as well as modern ones: [24,45].

Species names of vascular plants are consistent with the database *Plants of the World Online* [33], and those of algae are consistent with *AlgaeBase* [46]. We also consulted *Portale della flora d’Italia*, the web portal of Italian flora [47].

To determine the earliest recorded use of a plant species as a medicine, we consulted historical pharmaceutical works such as dispensatories, pharmacopoeias and pharmacy textbooks from 1600 to 1900. We cited the earliest known examples of medicinal use for each species mentioned (Section 3.4).

Plant species were identified using floras of the Mediterranean area by experienced florists.

## 5. Conclusions

The herbarium collection of Paulo Silvio Boccone, housed at Wrocław University in Poland, contains 142 numbered sheets with 312 terrestrial plant and marine organisms mounted on them. The specimens were collected mostly in today’s Italy during the author’s stay in Sicily and reflect his interest in searching for rare plant species, both wild and cultivated in gardens.

In the gardens, Boccone encountered at least seven exotic plants brought there as ornamentals that were later found to have medicinal value. This shows one of the ways of learning about exotic plants as sources of medicinal herbal material: from the garden to medicinal use (rather than from ethnopharmacological observations).

Boccone found *Narthecium reverchonii* Čelak, a very rare plant later recognised as endemic to Corsica.

Towards the end of his life, Boccone supported botanical research in Europe and the discovery of the medicinal properties of plants in his capacity as an editor, but he did not conduct any research on medicinal herbal materials himself.

## Data Availability

The original contributions presented in this study are included in the article. Further inquiries can be directed to the corresponding author.
